# Quantitative cardiovascular magnetic resonance: extracellular volume, native T1 and 18F-FDG PET/CMR imaging in patients after revascularized myocardial infarction and association with markers of myocardial damage and systemic inflammation

**DOI:** 10.1186/s12968-018-0454-y

**Published:** 2018-05-24

**Authors:** Karl P. Kunze, Ralf J. Dirschinger, Hans Kossmann, Franziska Hanus, Tareq Ibrahim, Karl-Ludwig Laugwitz, Markus Schwaiger, Christoph Rischpler, Stephan G. Nekolla

**Affiliations:** 1 Nuklearmedizinische Klinik und Poliklinik, Klinikum rechts der Isar, Technische Universität München, Ismaninger Straße 22, 81675 Munich, Germany; 2 Klinik und Poliklinik für Medizin I, Klinikum rechts der Isar, Technische Universität München, Ismaninger Straße 22, 81675 Munich, Germany; 3 DZHK (Deutsches Zentrum für Herz-Kreislauf-Forschung e.V.) partner site Munich Heart Alliance, Munich, Germany

**Keywords:** Myocardial infarction, T_1_ mapping, Inflammation, Extracellular volume, PET/MRI

## Abstract

**Background:**

Characterization of tissue integrity and inflammatory processes after acute myocardial infarction (AMI) using non-invasive imaging is predictive of patient outcome. Quantitative cardiovascular magnetic resonance (CMR) techniques such as native T_1_ and extracellular volume (ECV) mapping as well as ^18^F-FDG positron emission tomography (PET) imaging targeting inflammatory cell populations are gaining acceptance, but are often applied without assessing their quantitative potential. Using simultaneously acquired PET/CMR data from patients early after AMI, this study quantitatively compares these three imaging markers and investigates links to blood markers of myocardial injury and systemic inflammatory activity.

**Methods:**

A total of 25 patients without microvascular obstruction were retrospectively recruited. All imaging was simultaneously performed 5 ± 1 days after revascularization following AMI on an integrated 3T PET/MRI scanner. Native and post-contrast T_1_ data were acquired using a modified Look-Locker inversion recovery (MOLLI) sequence, ECV maps were calculated using individually sampled hematocrit. ^18^F-FDG PET was executed after 1 day of dietary preparation, 12 h of fasting, and administration of heparin. ECV, ^18^F-FDG and native T_1_ data were compared mutually as well as to peak counts of peripheral blood markers (creatine kinase, creatine kinase-MB, troponin, leukocytes, monocytes) and infarct size.

**Results:**

High intra-patient correlations of relative ECV, ^18^F-FDG PET and native T_1_ signal increases were observed in combination with no inter-patient correlation of maximum absolute values at the infarct center, suggesting well-colocalized but physiologically diverse processes begetting the respective image signals. Comparison of maximum image signals to markers of myocardial damage and systemic inflammation yielded highly significant correlations of ECV to peak creatine kinase-MB and overall infarct size as well as between native T_1_ and peak monocyte counts.

**Conclusions:**

Absolute native T_1_ values at the infarct core early after AMI can be linked to the systemic inflammatory response independent of infarct size. Absolute ECV at the infarct core is related to both infarct size and blood markers of myocardial damage.

**Electronic supplementary material:**

The online version of this article (10.1186/s12968-018-0454-y) contains supplementary material, which is available to authorized users.

## Background

The development of quantitative cardiovascular magnetic resonance (CMR) imaging techniques as a means for myocardial tissue characterization has seen a number of advances in recent years. Due to their relative robustness [1], especially extracellular volume (ECV) mapping and contrast media free native T_1_ mapping are being translated into clinical applications [2, 3] and both have shown promising results with respect to infiltrative and fibrotic cardiac diseases [4–6]. For acute myocardial infarction (AMI), equivalence or superiority of quantitative mapping approaches over qualitative techniques with respect to the delineation of the Area At Risk (AAR) [7, 8] and the prediction of functional outcome have been shown for native T_1_ [9, 10] and ECV [11] mapping. While there is ongoing discussion about the limits of detection for absolute changes of ECV in more subtle disease processes [12, 13], studies involving ECV and native T_1_ mapping often make only limited use of absolute values. Both mapping techniques have usually been evaluated as more sensitive or accurate versions of late gadolinium enhancement (LGE) or T_2_-weighted imaging for the determination of infarct size or AAR in the context of AMI [7, 8, 10, 11, 14]. This however neglects part of the quantitative potential of ECV and T_1_ mapping, which for the first time allow non-invasive CMR of the local severity of myocardial injury and edematous processes as opposed to only measuring their extent.

The assessment of post-AMI inflammatory processes have recently also come into the focus of positron emission tomography (PET) imaging, where ^18^F-fluorodeoxyglucose (^18^F-FDG) is used in combination with metabolic preparation to target cardiac infiltration by inflammatory cells [15, 16]. However, there is still uncertainty with respect to the contribution of infiltrative inflammatory cells and altered metabolism by post-ischemic but viable cardiomyocytes to the ^18^F-FDG imaging signal when applied in a clinical setting [15]. In this context, simultaneous PET/CMR imaging offers the potential for a deeper understanding of quantitative methods from both modalities and their relation to physiology [17, 18].

The study at hand employs PET/CMR to quantitatively investigate the relationship of the three imaging markers ^18^F-FDG uptake, native T_1_ and ECV in the context of a complex tissue state consisting of diverse processes including inflammation, edema and cellular tissue damage after revascularized AMI. In addition, blood markers of myocardial damage and blood counts of inflammatory cells have been obtained following the acute event. Consequently, quantitative regional results from the three imaging methods under investigation are compared among themselves as well as with peripheral blood parameters. It is investigated to what degree the three imaging methods indicate independent features of the post-ischemic healing process, and to what degree these features are co-localized.

Proceeding in this fashion, it is the goal of this manuscript to highlight the potential in making full use of available quantitative information from cardiac multimodality imaging for a better understanding of image signals and their relation to pathophysiology.

## Methods

### Patient cohort

Patients that were retrospectively enrolled for this investigation (*n* = 25) represent a large subgroup of a cohort from a previously published study [15], which has focused on global measures of ^18^F-FDG uptake and LGE and their relationship to functional outcome. All patients underwent examination on a clinical 3T PET/MRI scanner (Biograph mMR, Siemens Healthineers, Erlangen, Germany) 5 ± 1 days after myocardial infarction and subsequent, successful revascularization (Thrombolysis in Myocardial Infarction (TIMI) grade ≥ 2, average: 2.9). The study was approved by the local ethics committee, performed in agreement with the Declaration of Helsinki, and all participants gave written and informed consent. Criteria for retrospective enrollment were membership in the final study cohort reported in [15] and availability of native and post contrast T_1_ data. Additionally, patients showing signs of microvascular obstruction (MVO) in LGE images were excluded from quantitative analysis, as the necessary contrast agent equilibration for ECV mapping is not attainable. Also, segmentation of MVO border zones was deemed not practicable in these cases due to the differences in spatial resolution between PET and CMR images, and quantitative values would not have been comparable to results from transmural segmentation. A detailed description of the criteria for retrospective enrollment similar to the corresponding statements in [15] is given in Table 1.

**Table 1:** Criteria for Retrospective Enrollment

	n
Original cohort in [15]	49
Received ^18^F-FDG imaging and T_1_ mapping	45
Exclusion due to	n out of 45
Unsuccessful suppression of physiological ^18^F-FDG uptake	3
Previous infarctions revealed during imaging	2
Para-venous ^18^F-FDG injection	1
Acute pneumonia	1
Non-diagnostic T_1_ data	2
MVO at infarct core	11
Retrospectively enrolled	25

### PET imaging

^18^F-FDG PET was performed with patients receiving a low-carbohydrate diet the day before imaging, followed by a 12-h fasting period in order to suppress physiological myocardial FDG uptake. 30 min before ^18^F-FDG injection, patients received unfractionated heparin (50 UI/kg body weight intravenously) to further suppress physiological myocardial FDG uptake [15]. A list-mode PET scan in 3D mode was started 144 ± 39 min after intravenous injection of 311 ± 72 MBq of ^18^F-FDG. Correction of emission data was performed for randoms, scatter, dead time and attenuation. Attenuation correction was accomplished using 2-point Dixon sequence as previously described [19]. Parts of the body truncated in the attenuation map due to the limited field-of-view were recovered from PET emission data using the maximum likelihood reconstruction of attenuation and activity (MLAA) technique [20]. For reconstruction, a 3D attenuation-weighted ordered-subsets expectation maximization iterative reconstruction algorithm (AW-OSEM 3D) was used with three iterations and 21 subsets, Gaussian smoothing at 4 mm full width at half maximum, matrix size 344 × 344, zoom 1 and a resulting spatial resolution of 5 mm. For quantitative analysis, ^18^F-FDG image signals were expressed as standardized uptake values based on lean body mass (SUV LBM) or - for comparison to blood markers - also as tissue-to-background ratio (TBR) normalized to blood signal taken from an LV-centric region of interest.

### CMR imaging

As part of a comprehensive resting-state CMR exam, native and post-contrast T_1_ maps were acquired using a MOLLI prototype sequence in three short axis slices per patient. Acquisition (3(3)3(3)6) scheme [21]), retrospective motion correction and registration of native and post-contrast MOLLI data was performed as previously described [22]. If necessary, additional manual motion correction was applied to the T_1_ image series as it was acquired in shallow breathing. After registration, ECV maps were calculated from the resulting T_1_ maps using individually measured hematocrit. LGE images covering the complete left-ventricular (LV) myocardium were acquired directly following post contrast T_1_ maps after a cumulative dose of 0.2 mmol/kg Gd-DTPA (Magnevist, Bayer Healthcare, Berlin, Germany).

### PET/CMR image analysis

**Fig. 1:**
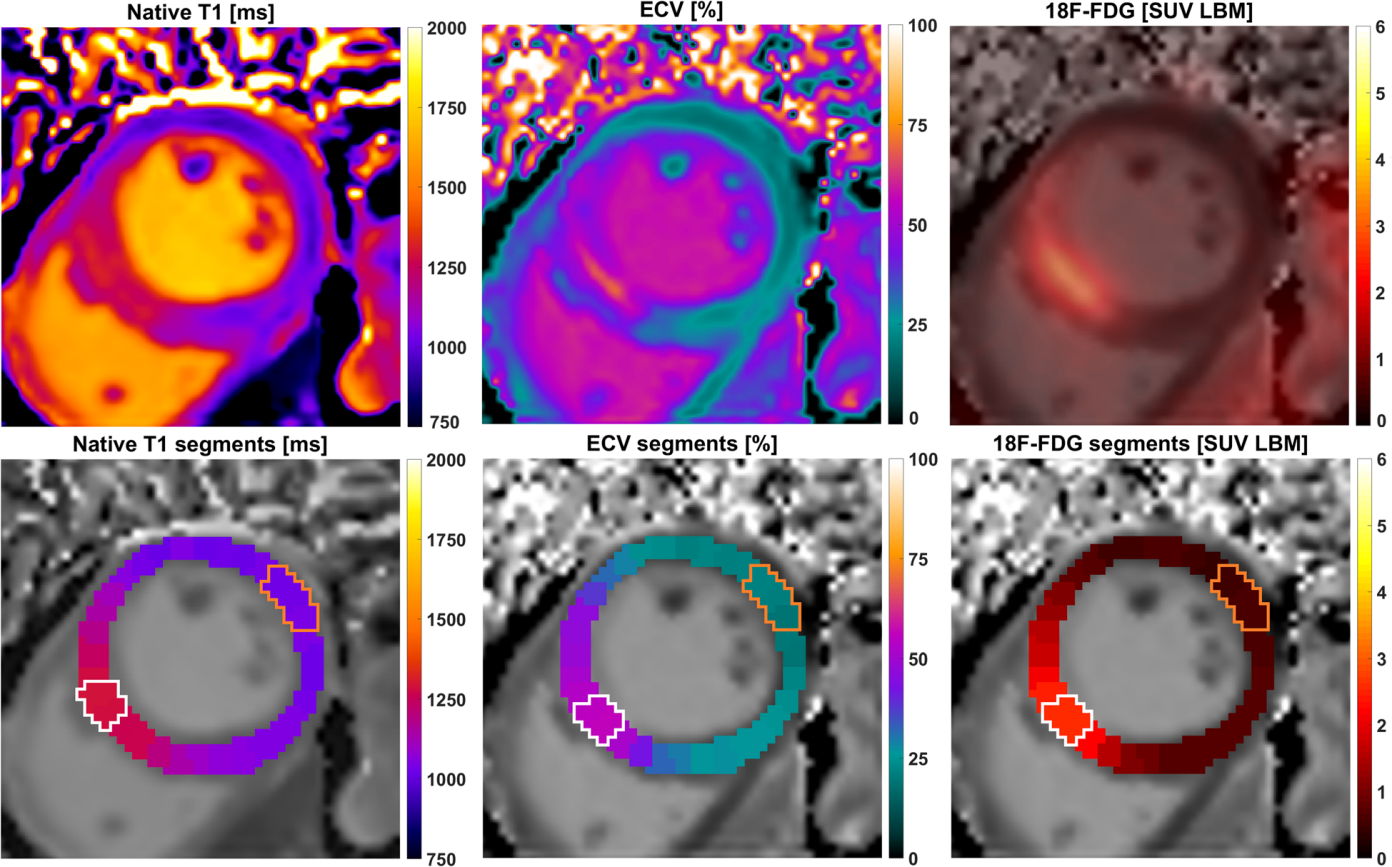
Visualization of the three analyzed image signals and their segmentation for one example case. The upper panel shows from left to right: native T_1_, extracellular volume and ^18^F-FDG uptake after fusion with the corresponding ECV map. The lower panel shows the respective segmentation results, consisting of 32 segments per slice that are co-localized between the three imaging methods. For each of these, white lines enclose the two sectors with the highest signal and orange lines the sectors defined as remote

For PET/CMR image registration, the MunichHeart software [23] was used to fuse ^18^F-FDG PET images with ECV and native T_1_ maps. Manual alignment was performed between ungated PET data and individually motion-corrected CMR data if necessary. Manual segmentation on the basis of ECV maps was performed for the most infarct-centric of the three acquired slices, which was determined on the basis of LGE images covering the whole left ventricle. Basal and mid-ventricular slices were segmented into 32 sectors (16 for apical slices), equally spaced along a centerline between endo and-epicardial borders without additional morphological reference. This segmentation was applied to the registered ECV, native T_1_ and ^18^F-FDG PET images of that slice. For each patient, segmentation of the chosen slice therefore resulted in 32 transmural sector values (16 for apical slices) for each of the three image signals under investigation as shown in Fig. 1. An average of the two highest sector values was taken as representing maximum insult severity for each patient and each image signal in the sense of an “imaging biopsy”. These maximum values were determined independently for each imaging method, and therefore do not necessarily refer to the exact same intra-slice location (see Fig. 1). For the quantitative analysis of remote regions, three sectors as distal as possible to the sector with the highest value were chosen manually. Therefore, for each patient, the remote value of ECV, native T1 and ^18^F-FDG uptake respectively refers to the average from the so-defined remote sectors, and the maximum value refers to the average of the two highest sector values. Additional measures of infarct size in % of LV volume were obtained by manual delineation of enhancement regions in the LGE images as described previously [15].

### Blood analysis

Daily blood sampling was performed for up to 6 days after revascularization. Peak levels of blood parameters creatine kinase (CK), creatine kinase-MB (CK-MB), troponin T as well as peak leukocyte and monocyte counts were taken from this sampling period as previously reported [15].

### Data analysis and statistics

Data processing and statistical analysis were executed in Matlab R2017a (Mathworks, Natick, Massachusetts, USA). Between-subject correlations represent a correlation of per-subject means, within-subject correlations were calculated using multiple regression (analysis of covariance). R- and *p*-values are given as Pearson correlation coefficients with a 5% significance level.

## Results

### Patient cohort

The physiological characteristics of the final patient cohort (*n* = 25) are shown in Table 2. A total of 42 individual segments from four patients were excluded before quantitative analysis due to small susceptibility artifacts in the T_1_ maps (*n* = 3) or breathing artifacts in the PET attenuation map (*n* = 1).

**Table 2:** Patient Characteristics

Characteristics	
Final [n] (%)	25 (100)
Male [n] (%)	22 (88)
Age [y]	66 ± 10
Pain to PCI [h]	7.2 ± 7.1
PCI to scan [d]	4.9 ± 1.4
HR at scan [bpm]	62 ± 9
Infarct size (LGE) [% LV]	17.3 ± 7.1
Avg. blood markers	
CK max [U/l]	1806 ± 930
CK-MB max [U/l]	211 ± 124
Troponin T max [U/l]	2.5 ± 1.7
Peak Leukocytes [G/l]	12.8 ± 4.3
Peak Monocytes [G/l]	1.1 ± 0.4

*CK* creatinine kinase, *LGE* late gadolinium enhancement, *PCI* percutaneous coronary intervention

### Mutual comparison of ^18^F-FDG, ECV and native T1

**Fig. 2:**
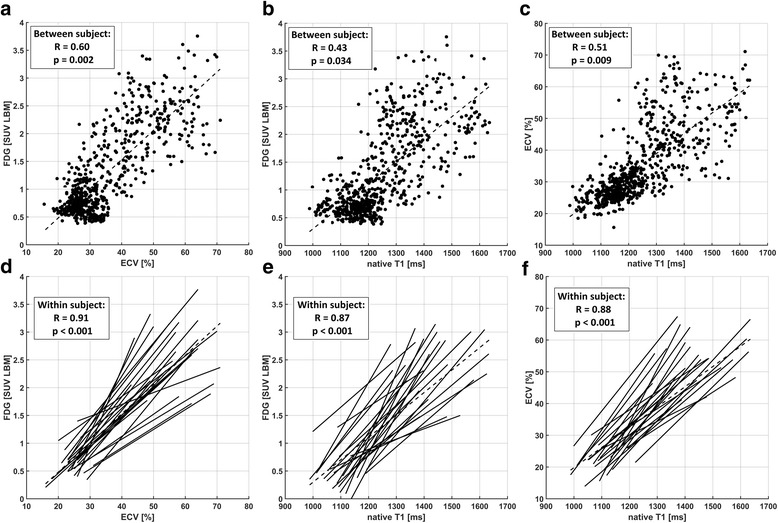
Pooled comparison (**a**-**c**) and individual regressions (**d**-**f**) between sector values from all three imaging methods. These are ^18^F-FDG vs. ECV (**a**/**d**), ^18^F-FDG vs. native T_1_ (b/e) and ECV vs. native T_1_ (c/f). Plots (a-c) each include results from all 662 sectors from all 25 patients and the correlation between subjects. Plots (**d**-**f**) visualize the different slopes of individual linear regressions and show the respective correlation factors within subjects

The total number of sectors after exclusions was 662 for each imaging method, consisting of 32 (mid/basal) or 16 (apical) sectors per patient for each of the 25 subjects. The upper panel of Fig. 2 shows the comparisons of these sector values in a globally pooled fashion. All three comparisons (FDG/ECV, FDG/native T1, ECV/native T1) exhibited similarly significant correlations between subject means (*R* = 0.60, 0.43, 0.51). The lower panel of Fig. 2 shows the corresponding within-patient correlations, i.e. a linearization of within-slice signal increase for each patient (i.e. each slice) individually. Within-patient analysis yielded much higher correlation factors (*R* = 0.91, 0.87, 0.88) than the globally pooled comparison.

**Fig. 3:**
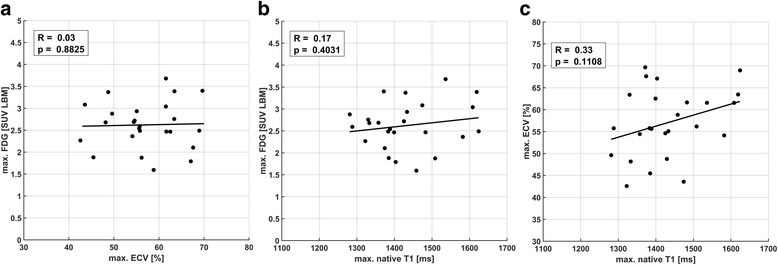
Comparison of maximum sector values between ECV/^18^F-FDG (**a**), native T1/^18^F-FDG (**b**) and ECV/native T1 (**c**) for all 25 patients, showing no significant correlations between the three methods. All points in plots (**a**-**c**) are equivalent to a per-patient average of the two highest sector values shown in the corresponding pooled comparisons in Fig. 2 (**a**-**c**)

The underlying inter-patient variability of absolute values at the high end was most clearly discernible for the comparison of FDG/ECV. Consequentially, if only the average of the two maximum sector values from each patient were compared, no significant correlations were observed for FDG/ECV, FDG/native T1 nor ECV/native T1 (Fig. 3). Table 3 shows ranges and averages for maximum and remote values across all patients for each imaging method. While maximum values were determined individually for each imaging method, average distances between sectors containing these were small across modalities, i.e. on average 1.2 sectors between ECV/FDG, 2 sectors between nT_1_/FDG and 1.9 sectors between nT_1_/ECV.

**Table 3:** Maximum and Remote Values for ECV, native T1 and ^18^F-FDG

	Mean	SD	Range
Max. ECV [%]	57.0	7.8	42.6–70.0
Maximum native T1 [ms]	1432	101	1281–1624
Max. FDG [SUV LBM]	2.62	0.53	1.62–3.74
Remote ECV [%]	27.8	3.9	20.3–34.7
Remote native T1 [ms]	1163	92	998–1419
Remote FDG [SUV LBM]	0.71	0.22	0.45–1.22

*ECV* extracellular volume, *FDG* fluorodeoxyglucose, *SUV LBM* standardized uptake values based on lean body mass

### Comparison of maximum image signals and infarct size

**Fig. 4:**
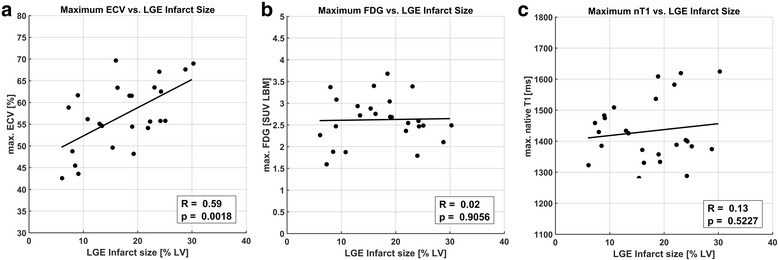
Comparison of maximum ECV, ^18^F-FDG uptake and native T_1_ values from the most infarct-centric slice with relative global infarct size as measured independently from LGE images in % LV. A good correlation between infarct extent and maximum tissue damage in terms of ECV was observed (**a**), while no correlation was seen between infarct size and maximum ^18^F-FDG uptake (**b**) and native T_1_ (**c**)

Results for comparing infarct sizes with corresponding maximum values of ECV, ^18^F-FDG uptake and native T_1_ are shown in Fig. 4. Infarct sizes calculated on the basis of LGE images yielded an average infarct size of 17.3 ± 7.1% of LV volume. A highly significant correlation of cellular damage as indicated by maximum ECV with infarct size was observed (*p* = 0.002). Conversely, no significant correlation between infarct size and either maximum ^18^F-FDG uptake or maximum native T_1_ was observed.

### Comparison of maximum image signals and blood markers

**Fig. 5:**
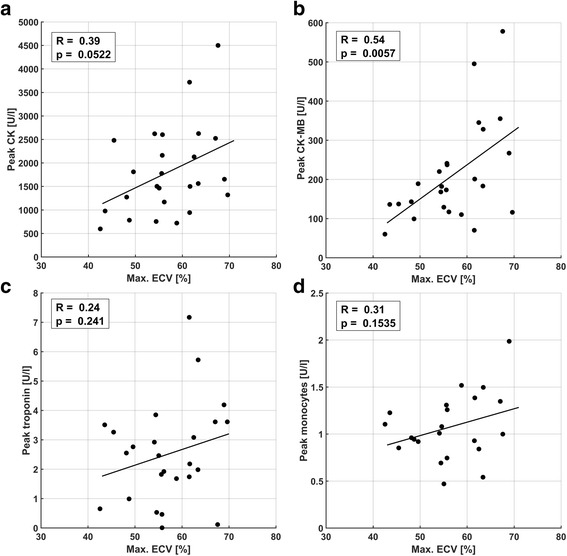
Comparison of maximum ECV with peak blood parameters CK (**a**), CK-MB fraction (**b**), troponin (**c**), and monocyte counts (**d**). A narrowly insignificant trend for CK (*p* = 0.052) and a highly significant correlation to CK-MB (*p* = 0.0057) were observed

**Fig. 6:**
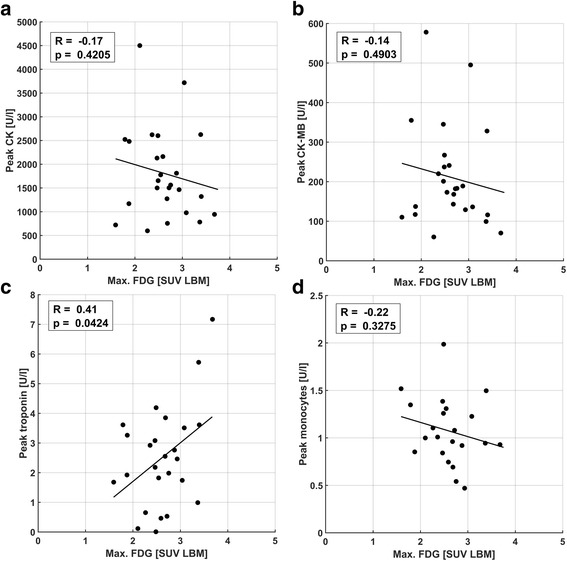
Comparison of maximum ^18^F-FDG uptake with peak blood parameters CK (**a**), CK-MB fraction (**b**), troponin (**c**), and monocyte counts (**d**). A narrowly significant trend for troponin (*p* = 0.042) was observed

**Fig. 7:**
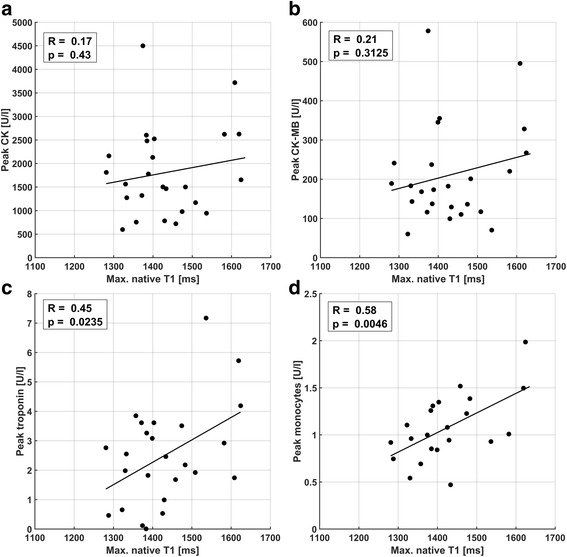
Comparison of maximum native T_1_ with peak blood parameters CK (**a**), CK-MB fraction (**b**), troponin (**c**), and monocyte counts (**d**). A narrowly significant trend for troponin (*p* = 0.024) and a highly significant correlation (*p* = 0.0046) for monocyte counts was found. No significant correlations to CK or CK-MB fraction were observed

Maximum ECV (Fig. 5), ^18^F-FDG uptake (absolute (Fig. 6) and TBR) and native T_1_ (Fig. 7) were compared to peak values of peripheral blood parameters CK, CK-MB, troponin T as well as peak leukocyte and monocyte counts. Monocyte counts were unavailable for two patients, and were excluded for one additional patient due to splenectomy, resulting in 22 instead of 25 data points for Figs. 5d, 6d, 7d. For maximum ECV, a strong trend towards association with peak CK (*p* = 0.052) and a significant correlation with peak CK-MB (*p* = 0.006) were observed, despite no correlation to peak troponin or monocytes. The comparison to peak CK-MB revealed a tight relationship at the low end and a larger variability at the high end of values (Fig. 5b). For absolute ^18^F-FDG uptake, only a narrowly significant correlation was found with troponin (*p* = 0.042), and none was found for ^18^F-FDG TBR normalized to LV blood (see Additional File 1). Maximum native T_1_ values did not show significant correlations to CK or CK-MB, but a highly significant correlation (*p* = 0.005) to peak monocyte counts and a significant correlation with troponin (*p* = 0.024). The corresponding comparisons to peak leukocyte counts are not shown, yielding a significant trend (*p* = 0.033) with native T_1_.

## Discussion

The study at hand has compared absolute measures of ^18^F-FDG uptake, extracellular volume and native T_1_ in patients early after revascularized myocardial infarction using simultaneously acquired PET/CMR data. Quantitative results have been derived for a single, infarct-centric slice position that was co-localized between all three methods, which can be seen as a biopsy-like imaging approach. An effort was made to link the so-obtained image signals to underlying pathophysiological processes using independent measures of infarct size and peripheral blood markers of cardiac damage and inflammatory cell populations. The study did not investigate correlations between image signals and functional recovery post AMI as these have already been given separately for ECV [11], ^18^F-FDG [15] and native T_1_ [9] in quantitative or semi-quantitative fashion.

### Comparison of signal localization and magnitude

The first step was the comparison of inter-patient and intra-patient (i.e. intra-slice) signals as shown in Fig. 2. The excellent correlations for the latter suggest a very good co-localization of pathophysiological processes indicated by the three different image signals. While in the vast majority of studies, extents of image signals are compared by global thresholding, e.g. using multiples of standard deviations as binary cutoff values, the intra-patient correlations shown herein are independent of differences in absolute signal increase between patients, i.e. differences seen in the correlation slopes in Fig. 2c-d. Despite the good co-localization of signal increases, the heterogeneity of these slopes and equivalently the comparison of maximum values in Fig. 3 suggest sensitivities to different underlying tissue properties. Therefore, ECV, ^18^F-FDG uptake and native T_1_ appeared as representing mutually distinct combinations of features with respect to infarct-related pathophysiology, while the respective underlying processes seemed to be largely co-localized.

### Comparison of signal magnitudes and external markers

For myocardial ECV, estimates derived from pre- and post-contrast T_1_ mapping have been shown to be sensitive to a number of different disease processes [5] and histologically verified to correlate with fibrosis [24]. With respect to AMI, quantitative ECV mapping has only recently been shown to be associated with functional outcome in patients [11] but further investigation of pathophysiological mechanisms are lacking. While significant association of ECV with edema has been documented at day 1 after AMI in pigs [25], the same study reported a disappearance of this association after 7 days, which suggests edema if at all as a minor contributor to ECV estimates from the study at hand obtained 5 days after AMI. A possible pathophysiological correlate for absolute ECV in this study has been provided in the form of CK/CK-MB blood markers for (myocytic) cellular damage. The significant correlation between peak CK-MB and ECV is in fact remarkable as it reflects the association of a global, peripheral blood parameter with a focal, biopsy-like imaging result. It may however be seen as an epiphenomenon to the additional finding of a highly significant correlation between ECV at the infarct center and infarct size, where peak CK-MB activity is an established marker for the latter [26]. This observed relationship between the extent of the area being subject to ischemic insult and the amount of myocytic damage at its center may be seen as somewhat mechanistically plausible, considering a decrease of the probability for remaining collateralization with distance to the nearest non-infarcted tissue regions. The much smaller significance for the corresponding correlation observed between peak CK and ECV is consistent with a lower specificity of CK to myocardial damage compared to CK-MB.

With respect to FDG, data from this study suggest no correlation of maximum ^18^F-FDG uptake at the infarct center with peak monocyte or leukocyte counts or global infarct size. This was irrespective of whether maximum ^18^F-FDG was evaluated in absolute terms as SUV LBM or normalized to LV blood activity as a TBR. A similar finding [15] has been interpreted as a conceivable disproportionality between systemic/peripheral inflammatory cell counts and the presence of migrated inflammatory cells within the myocardium begetting the imaging signal. While it is known that ^18^F-FDG is taken up by inflammatory cell populations [27], the interpretation of a corresponding image signal from the post-ischemic myocardium is challenging due to the concurrent presence of background contributions from myocyte uptake. Despite a somewhat reliable suppression of physiologic FDG metabolism in healthy cardiomyocytes, the potential presence of post-ischemic FDG uptake due to a switch of metabolism from fatty acids towards glucose consumption early after AMI is a major confounder to the interpretation of ^18^F-FDG uptake as a purely inflammatory signal [28]. Therefore, ^18^F-FDG image signals are generally regarded as a mixture of background/blood pool, post-ischemic and inflammatory constituents in this context.

Native myocardial T_1_ may reflect a variety of pathologic tissue alterations, but is generally accepted to indicate the edematous increase of free water content early after AMI [8]. The expected increase of infarct-centric native T_1_ observed in this study is therefore attributed to an edematous reaction, which, however, did not show a correlation with infarct size as ECV did. With respect to native T_1_, the most interesting finding from this study is a highly significant correlation with peak monocyte counts and a weaker but still significant association to peak leukocyte counts, of which monocytes are a subset specific to inflammatory activity. As for ECV, this association of biopsy-like imaging results with peripheral blood markers is remarkable, even more so considering that native T_1_ was not found to be related to infarct size. Therefore, the data at hand provides evidence for the fact that myocardial edema and the systemic inflammatory reaction post AMI are quantitatively associated.

Summarizing the comparison of imaging results with peripheral blood parameters, the data at hand suggest ECV as a marker of cellular damage early after reperfused AMI, with maximum values related to infarct size and therefore reflecting most likely a mechanistic property of the respective infarct. Conversely, the missing correlation to infarct size for maximum ^18^F-FDG uptake and native T_1_ suggest their association with a more patient- than infarct-specific reaction to the ischemic insult. This notion is strongly supported by the observed association of native T_1_ with peak monocyte counts.

### Limitations

An important translational limitation to results from this study is that patients exhibiting MVO post AMI were not amenable to the presented analysis. While there exist propositions on the segmentation of MVO border zones for e.g. ECV mapping [11], the difference in spatial resolution between PET and CMR precluded comparable sub-segmentations of the myocardial wall. For similar reasons, only transmural short-axis sectors were defined for LV myocardium, where especially cardiac motion hampers a meaningful sub-segmentation across the myocardial wall in PET. With respect to segmentation, locations for maximum values were determined individually for each modality because the alternative of having one of the three methods be the reference standard for locating the corresponding sectors would have introduced a bias into the comparison and additionally precluded the translation of conclusions to situations where the reference modality is not available. The small spatial differences introduced by individual determination of reference sectors did not suggest this as a significant limitation to the finding that all three modalities indicate mutually distinct processes within the tissue.

The acquisition scheme (3(3)3(3)6) used in this study for MOLLI T_1_ mapping has been shown to be more sensitive to variations in heart rate than other, more recently proposed schemes [29]. However, the fact that resting heart rates in the examined cohort did not vary strongly and that heart rate did not correlate with remote native T_1_ (*R* = − 0.22, *p* = 0.3) do not suggest this as a major confounder to the presented findings.

Additionally, the practice of using peak values of peripheral blood markers as a surrogate for their summed activity may have introduced additional variability into the reported results. While the described practice is known to be relatively accurate for CK/CK-MB [26], the inflammatory reaction indicated by monocyte counts may behave in a more complex fashion. With respect to statistics, the large number of performed correlation analyses may have justified the use of a 1% significance level to make type 1 errors less likely. However, the main conclusions presented herein only rely on findings with *p*-values < 0.01.

## Conclusions

Simultaneously acquired PET/CMR data from this study have shown a close spatial concordance of relative signal increase in combination with a divergence of absolute signal magnitudes between ^18^F-FDG uptake, native T_1_ and ECV early after revascularized AMI. A biopsy-like imaging approach has revealed links between CMR-derived ECV estimates and blood markers of muscular damage as well as an association of the edematous response indicated by absolute native T_1_ estimates with the systemic inflammatory activity indicated by peripheral monocyte counts.

## Additional file

Additional file 1: Comparison of 18F-FDG uptake TBR (normalized to LV blood activity) with peripheral blood markers CK, CK-MB, troponin and monocyte counts. (PNG 568 kb)

## Abbreviations

AAR: Area at risk
AMI: Acute myocardial infarction
AW-OSEM: Attenuation-weighted ordered-subsets expectation maximization
CK: Creatine kinase
CK-MB: Creatine kinase-MB
CMR: Cardiovascular magnetic resonance
ECV: Extracellular volume
FDG: Fluorodeoxyglucose
Gd-DTPA: Gadopentetic acid
LGE: Late gadolinium enhancement
LV: Left ventricular/left ventricle
MLAA: Maximum likelihood reconstruction of attenuation and activity
MOLLI: Modified Look-Locker inversion recovery
MVO: Microvascular obstruction
PET: Positron emission tomography
SUV LBM: Standardized uptake value based on lean body mass
TBR: Tissue-to-background ratio
TIMI: Thrombolysis in myocardial infarction

## References

[1] Kellman P, Arai AE, Xue H (2013). T1 and extracellular volume mapping in the heart: estimation of error maps and the influence of noise on precision. J Cardiovasc Magn Reson.

[2] Moon JC, Messroghli DR, Kellman P, Piechnik SK, Robson MD, Ugander M, Gatehouse PD, Arai AE, Friedrich MG, Neubauer S, Schulz-Menger J, Schelbert EB (2013). Myocardial T1 mapping and extracellular volume quantification: a Society for Cardiovascular Magnetic Resonance (SCMR) and CMR working Group of the European Society of cardiology consensus statement. J Cardiovasc Magn Reson.

[3] White SK, Sado DM, Flett AS, Moon JC (2012). Characterising the myocardial interstitial space: the clinical relevance of non-invasive imaging. Heart.

[4] Greulich S, Kitterer D, Latus J, Aguor E, Steubing H, Kaesemann P, Patrascu A, Greiser A, Groeninger S, Mayr A, Braun N, Alscher MD, Sechtem U, Mahrholdt H (2016). Comprehensive cardiovascular magnetic resonance assessment in patients with sarcoidosis and preserved left ventricular ejection fraction. Circ Cardiovasc Imaging.

[5] Sado DM, Flett AS, Banypersad SM, White SK, Maestrini V, Quarta G, Lachmann RH, Murphy E, Mehta A, Hughes DA, McKenna WJ, Taylor AM, Hausenloy DJ, Hawkins PN, Elliott PM, Moon JC (2012). Cardiovascular magnetic resonance measurement of myocardial extracellular volume in health and disease. Heart.

[6] Kellman P, Wilson JR, Xue H, Bandettini WP, Shanbhag SM, Druey KM, Ugander M, Arai AE (2012). Extracellular volume fraction mapping in the myocardium, part 2: initial clinical experience. J Cardiovasc Magn Reson.

[7] Bulluck H, White SK, Rosmini S, Bhuva A, Treibel TA, Fontana M, Abdel-Gadir A, Herrey A, Manisty C, Wan SMY, Groves A, Menezes L, Moon JC, Hausenloy DJ (2015). T1 mapping and T2 mapping at 3T for quantifying the area-at-risk in reperfused STEMI patients. J Cardiovasc Magn Reson.

[8] Ugander M, Bagi PS, Oki AJ, Chen B, Hsu LY, Aletras AH, Shah S, Greiser A, Kellman P, Arai AE (2012). Myocardial edema as detected by pre-contrast T1 and T2 CMR delineates area at risk associated with acute myocardial infarction. JACC Cardiovasc Imaging.

[9] Dall'Armellina E, Piechnik SK, Ferreira VM, Si QL, Robson MD, Francis JM, Cuculi F, Kharbanda RK, Banning AP, Choudhury RP, Karamitsos TD, Neubauer S (2012). Cardiovascular magnetic resonance by non contrast T1-mapping allows assessment of severity of injury in acute myocardial infarction. J Cardiovasc Magn Reson.

[10] Ferreira VM, Piechnik SK, Dall'Armellina E, Karamitsos TD, Francis JM, Choudhury RP, Friedrich MG, Robson MD, Neubauer S (2012). Non-contrast T1-mapping detects acute myocardial edema with high diagnostic accuracy: a comparison to T2-weighted cardiovascular magnetic resonance. J Cardiovasc Magn Reson.

[11] Kidambi A, Motwani M, Uddin A, Ripley DP, McDiarmid AK, Swoboda PP, Broadbent DA, Musa TA, Erhayiem B, Leader J, Croisille P, Clarysse P, Greenwood JP, Plein S (2017). Myocardial extracellular volume estimation by CMR predicts functional recovery following acute MI. JACC Cardiovasc Imaging.

[12] Carberry J, Carrick D, Haig C, Rauhalammi SM, Ahmed N, Mordi I, McEntegart M, Petrie MC, Eteiba H, Hood S, Watkins S, Lindsay M, Davie A, Mahrous A, Ford I, Sattar N, Welsh P, Radjenovic A, Oldroyd KG, Berry C (2016). Remote zone extracellular volume and left ventricular remodeling in survivors of ST-elevation myocardial infarction. Hypertension.

[13] Treibel TA, Zemrak F, Sado DM, Banypersad SM, White SK, Maestrini V, Barison A, Patel V, Herrey AS, Davies C, Caulfield MJ, Petersen SE, Moon JC (2015). Extracellular volume quantification in isolated hypertension - changes at the detectable limits?. J Cardiovasc Magn Reson.

[14] Messroghli DR, Walters K, Plein S, Sparrow P, Friedrich MG, Ridgway JP, Sivananthan MU (2007). Myocardial T1 mapping: application to patients with acute and chronic myocardial infarction. Magn Reson Med.

[15] Rischpler C, Dirschinger RJ, Nekolla SG, Kossmann H, Nicolosi S, Hanus F, van Marwick S, Kunze KP, Meinicke A, Götze K, Kastrati A, Langwieser N, Ibrahim T, Nahrendorf M, Schwaiger M, Laugwitz KL. Prospective evaluation of 18F-Fluorodeoxyglucose uptake in Postischemic myocardium by simultaneous positron emission tomography/magnetic resonance imaging as a prognostic marker of functional outcome. Circulation Cardiovascular imaging 2016;9:e004316. 10.1161/CIRCIMAGING.115.004316 PMC4826716 27056601

[16] Wollenweber T, Roentgen P, Schäfer A, Schatka I, Zwadlo C, Brunkhorst T, Berding G, Bauersachs J, Bengel FM (2014). Characterizing the inflammatory tissue response to acute myocardial infarction by clinical multimodality noninvasive imaging. Circ Cardiovasc Imaging.

[17] Rischpler C, Nekolla SG, Kunze KP, Schwaiger M (2015). PET/MRI of the heart. Semin Nucl Med.

[18] Schwaiger M, Kunze K, Rischpler C, Nekolla SG (2017). PET/MR: yet another tesla?. J Nucl Cardiol.

[19] Martinez-Möller A, Souvatzoglou M, Delso G, Bundschuh RA, Chefd'hotel C, Ziegler SI, Navab N, Schwaiger M, Nekolla SG (2009). Tissue classification as a potential approach for attenuation correction in whole-body PET/MRI: evaluation with PET/CT data. J Nucl Med.

[20] Nuyts J, Bal G, Kehren F, Fenchel M, Michel C, Watson C (2013). Completion of a truncated attenuation image from the attenuated PET emission data. IEEE Trans Med Imaging.

[21] Messroghli DR, Radjenovic A, Kozerke S, Higgins DM, Sivananthan MU, Ridgway JP (2004). Modified look-locker inversion recovery (MOLLI) for high-resolution T1 mapping of the heart. Magn Reson Med.

[22] Kunze KP, Rischpler C, Hayes C, Ibrahim T, Laugwitz K-L, Haase A, Schwaiger M, Nekolla SG (2017). Measurement of extracellular volume and transit time heterogeneity using contrast-enhanced myocardial perfusion MRI in patients after acute myocardial infarction. Magn Reson Med.

[23] Metz S, Ganter C, Lorenzen S, van Marwick S, Herrmann K, Lordick F, Nekolla SG, Rummeny EJ, Wester H-J, Brix G, Schwaiger M, Beer AJ (2010). Phenotyping of tumor biology in patients by multimodality multiparametric imaging: relationship of microcirculation, avb3 expression, and glucose metabolism. J Nucl Med.

[24] Diao K-Y, Yang Z-G, Xu H-Y, Liu X, Zhang Q, Shi K, Jiang L, Xie L-J, Wen L-Y, Guo Y-K (2017). Histologic validation of myocardial fibrosis measured by T1 mapping: a systematic review and meta-analysis. J Cardiovasc Magn Reson.

[25] Jablonowski R, Engblom H, Kanski M, Nordlund D, Koul S, Van Der Pals J, Englund E, Heiberg E, Erlinge D, Carlsson M, Arheden H (2015). Contrast-enhanced CMR overestimates early myocardial infarct size: mechanistic insights using ECV measurements on day 1 and day 7. JACC Cardiovasc Imaging.

[26] Fiolet JW, ter Welle HF, van Capelle FJ, Lie KI (1983). Infarct size estimation from serial CK MB determinations: peak activity and predictability. Br Heart J.

[27] Lee WW, Marinelli B, Van Der Laan AM, Sena BF, Gorbatov R, Leuschner F, Dutta P, Iwamoto Y, Ueno T, Begieneman MPV, Niessen HWM, Piek JJ, Vinegoni C, Pittet MJ, Swirski FK, Tawakol A, Di Carli M, Weissleder R, Nahrendorf M (2012). PET/MRI of inflammation in myocardial infarction. J Am Coll Cardiol.

[28] Schwaiger M, Schelbert HR, Ellison D, Hansen H, Yeatman L, Vinten-Johansen J, Selin C, Barrio J, Phelps ME (1985). Sustained regional abnormalities in cardiac metabolism after transient ischemia in the chronic dog model. J Am Coll Cardiol.

[29] Kellman P, Hansen MS (2014). T1-mapping in the heart: accuracy and precision. J Cardiovasc Magn Reson.

