# Supplementary figures and images for: Quantitative cardiovascular magnetic resonance: extracellular volume, native T1 and 18F-FDG PET/CMR imaging in patients after revascularized myocardial infarction and association with markers of myocardial damage and systemic inflammation
Source: J Cardiovasc Magn Reson. 2018 May 24;20:33. doi: 10.1186/s12968-018-0454-y (PMC5967072; doi:10.1186/s12968-018-0454-y)

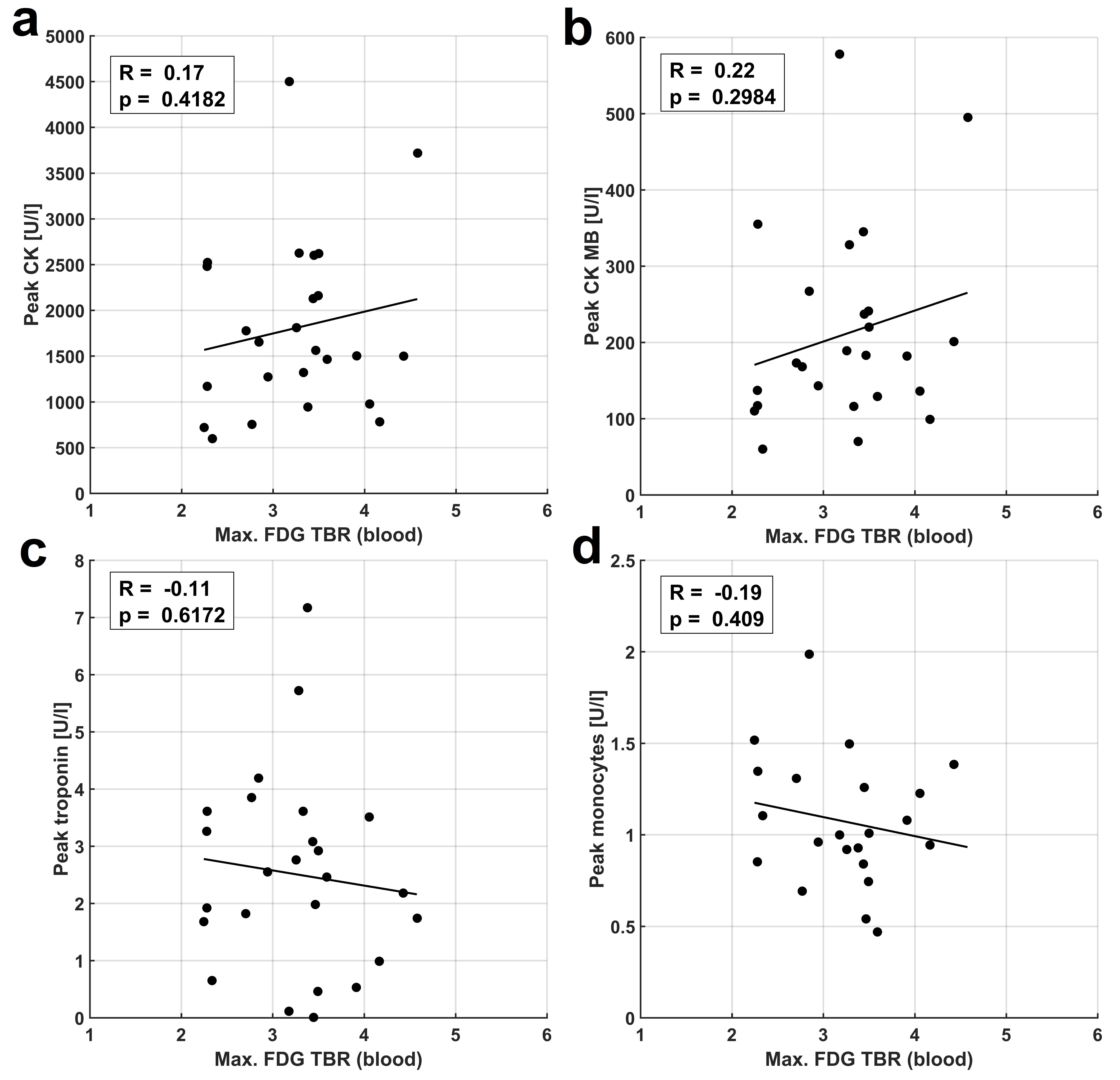

Supplement: Supplementary file 1 — Comparison of 18F-FDG uptake TBR (normalized to LV blood activity) with peripheral blood markers CK, CK-MB, troponin and monocyte counts. (PNG 568 kb) [file 12968_2018_454_MOESM1_ESM.png]
